# Supplementary material for: Immune Response Modulation by HPV16 Oncoproteins in Lung Cancer: Insights from Clinical and In Vitro Investigations
Source: Viruses. 2024 Nov 4;16(11):1731. doi: 10.3390/v16111731 (PMC11599038; doi:10.3390/v16111731)
Supplement: Supplementary file 1 [file viruses-16-01731-s001.zip › viruses-3229813-supplementary.pdf]

Suplementary Materials for

Immune Response Modulation by HPV16 Oncoproteins in Lung Cancer: Insights from Clinical and In Vitro Investigations.

Table S1: Primers used to detect the expression of HPV16 genes

Real Time PCR primer	Sequence (5'-3')
E2 HPV 16 F	AACGAAGTATCCTCTCCTGAAATTATTAG
E2 HPV 16 R	CCAAGGCGACGGCTTTG
E5 HPV 16 F	ACTGGCTGCTTTTTGCTTTG
E5 HPV16 R	GACACAGACAAAAGCAGCGG
E6 HPV16 F	TGCAATGTTTCAGGACCC
E6 HPV16 R	CATAACTGTGTGGTAACTTTCTGGG
E7 HPV16 F	AGCTCAGAGGAGGAGGATGA
E7 HPV16 R	GAGACCAGATGGGGCACACA
ACTB F	AAGAGAGGCATCCTCACCCT
ACTB R	TACATGGCTGGGGTGTTGAA
EEF1A1 F	GTTGCGGTGGGTGTCATCA
EEF1A1 R	GAGTGGGGTGGCAGGTAT

E5 - Ct Curve

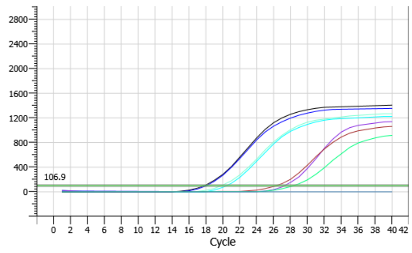

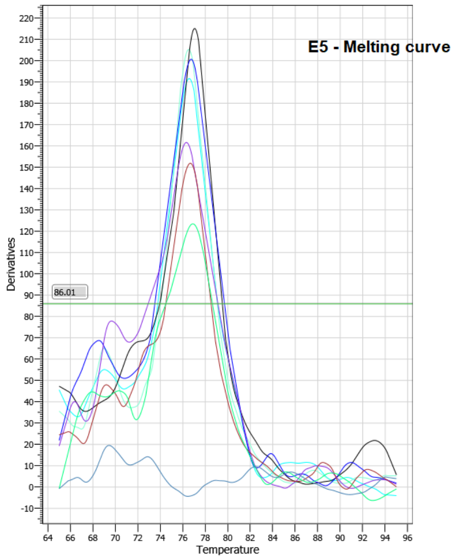

E6 - Ct Curve

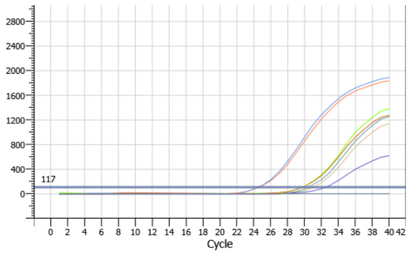

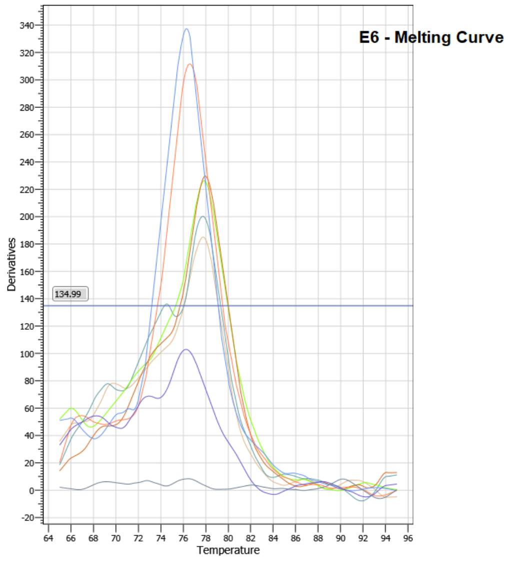

E7 - Ct Curve

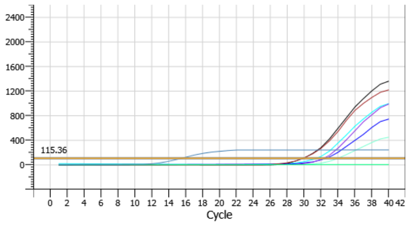

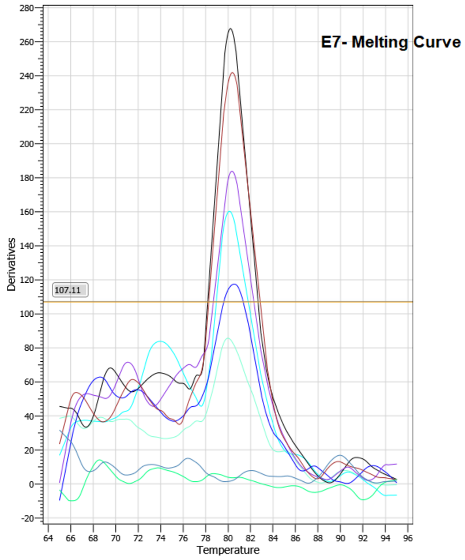

Figure S1. Confirmation of oncoproteins transfection in a cell line model. Ct and Melting curve representing the expression of oncogenes E5, E6, and E7 transfected in A549

Table S2. Clinical-Demographic Characteristics Frequencies

Characteristics	Frequency (%)
**Gender**	
Male	12 (66.7)
Female	6 (33.3)
**Age**	
11 - 55.	6 (33.3)
56 - 81	12 (66.7)
**Smoking**	
Yes	14 (77.8)
No	4 (22.2)
**Alcohol Consumption**	
Yes	11 (61.1)
No	7 (38.9)
**Presence of HPV**	
Yes	13 (72.2)
No	5 (27.8)

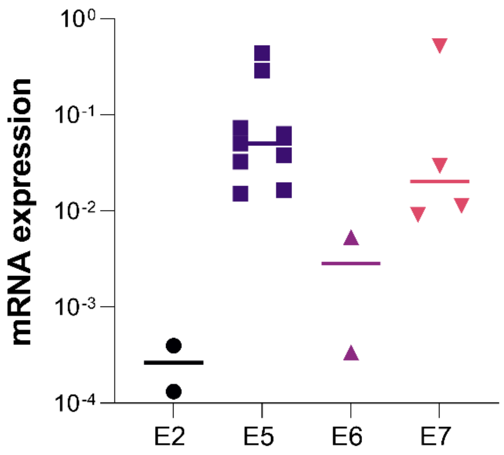

Figure S2. Individual mRNA expression values of HPV16 oncogenes in tumors from lung cancer patients. The expression of the E2 gene assessed in this study was compared with the expression of the oncogenes E5, E6, and E7, which were analyzed in previous studies published by our group (São Marcos et la., 2022). No statistically significant difference was found.

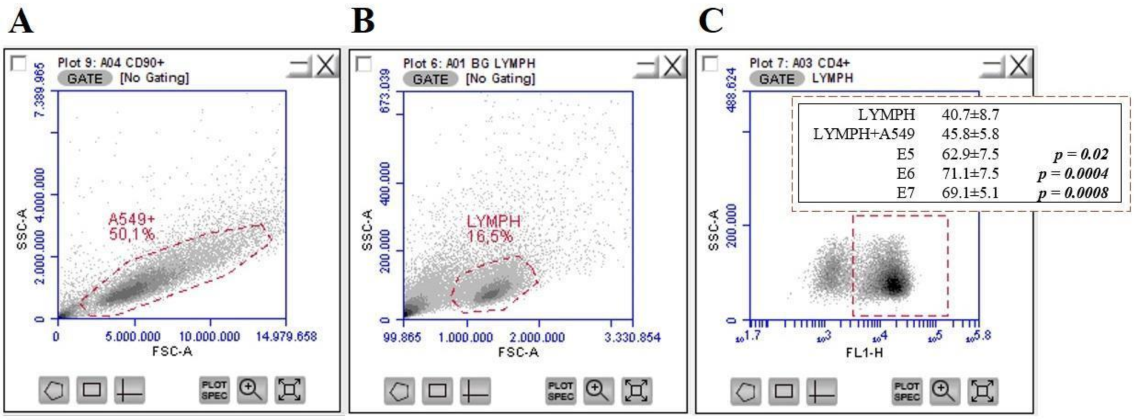

Figure S3. Density plots representation. **A** – background of A549 cells ($CD90^+$). **B** - background of lymphocytes ($CD3^+$). **C** - background of CD4+ lymphocytes (CD3+CD4+) and MEAN±SD of each experimental group. The *p* values were obtained comparing the group LYMPH+A549 with E5, E6, and E7, respectively.

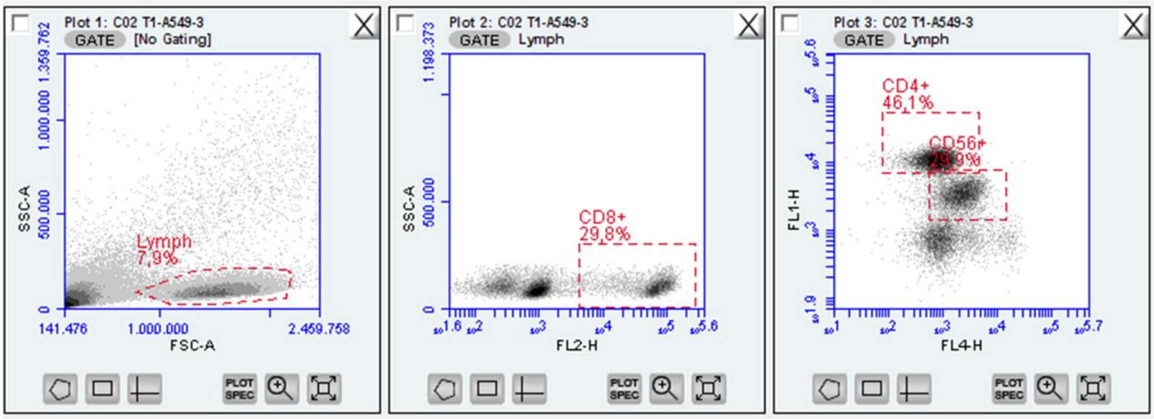

Figure S4. The gating strategy used to identify different lymphocyte subpopulations. Within the lymphocyte gate, CD8+ T cells were stained with PE and detected using the FL2-H channel. CD4+ T cells (stained with FITC) and CD56+ natural killer (NK) cells (stained with APC) were distinguished using the FL1-H (CD4) and FL4-H (CD56) channels, respectively. This gating strategy was consistently applied to all samples in the study to accurately identify and quantify the relevant lymphocyte subsets.

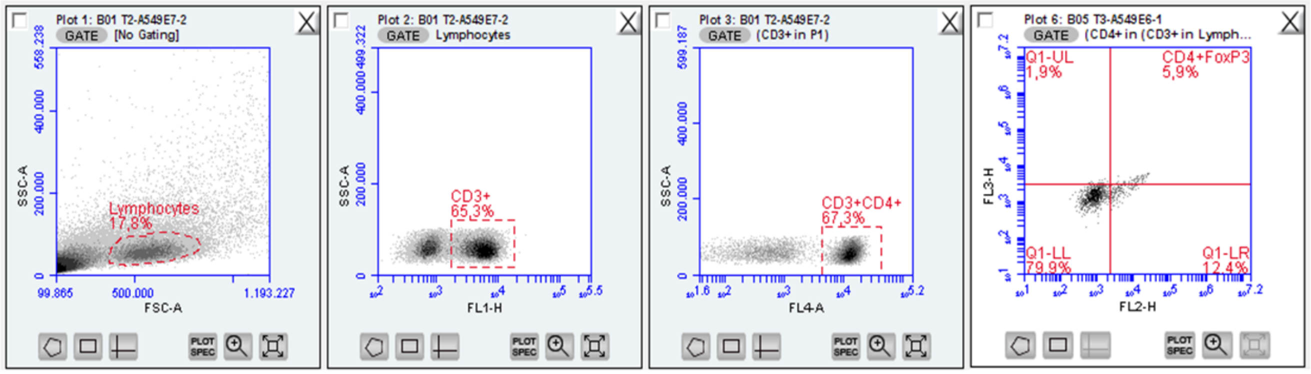

Figure S5. The gating strategy used to identify regulatory T cells (Tregs). CD3+ T cells were identified using FITC fluorescence in the FL1-H channel, and CD4+ T cells were gated within the CD3+ population using APC fluorescence in the FL4-H channel. Further gating of CD4+ T cells was performed to identify regulatory T cells (Tregs), characterized by the expression of CD25 and FOXP3. CD25 was labeled with PerCP (FL3-H), and FOXP3 was labeled with PE (FL2-H).

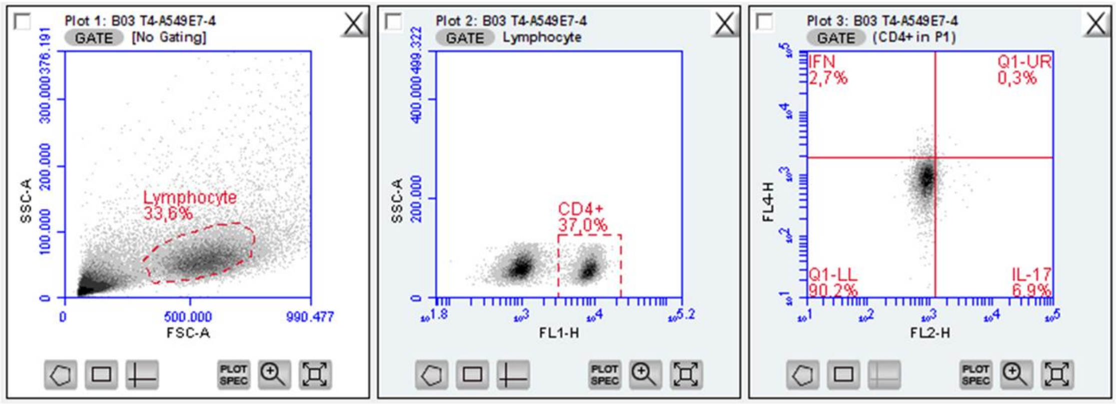

Figure S6. The gating strategy to identify CD4+ T cells and cytokine IL-17 and IFN production. CD4+ T cells were identified within the lymphocyte population using FITC fluorescence in the FL1-H channel. IL-17 (labeled with PE, FL2-H) and IFN-γ (labeled with APC, FL4-H) were measured. The quadrant statistics show the CD4+ population producing IFN-γ and IL-17.

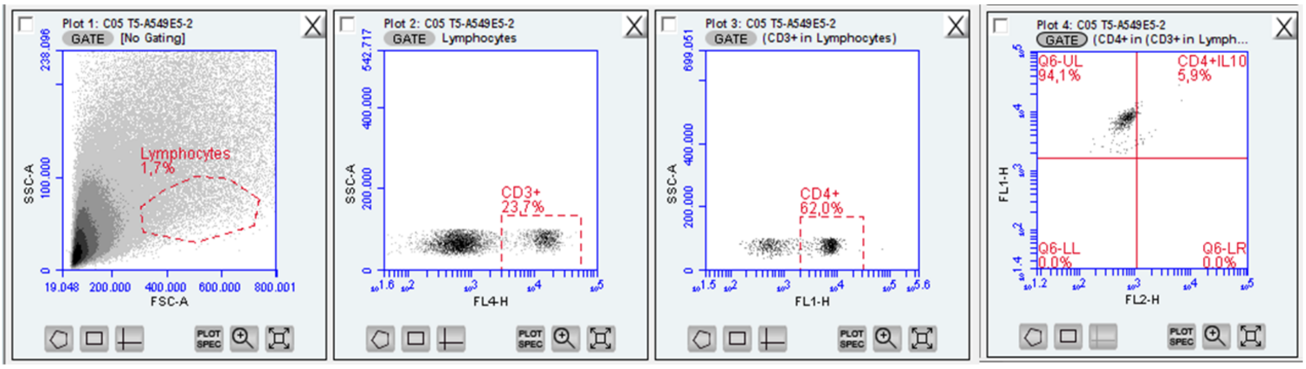

Figure S7. The gating strategy to identify IL-10 expression in CD4+ T lymphocytes. CD3+ T cells were first identified using APC staining in the FL4-H channel. CD4+ T cells were then gated within the CD3+ population, identified by FITC staining in the FL1-H channel. Finally, IL-10 expression was analyzed within the CD4+ subset, using PE staining detected in the FL2-H channel.

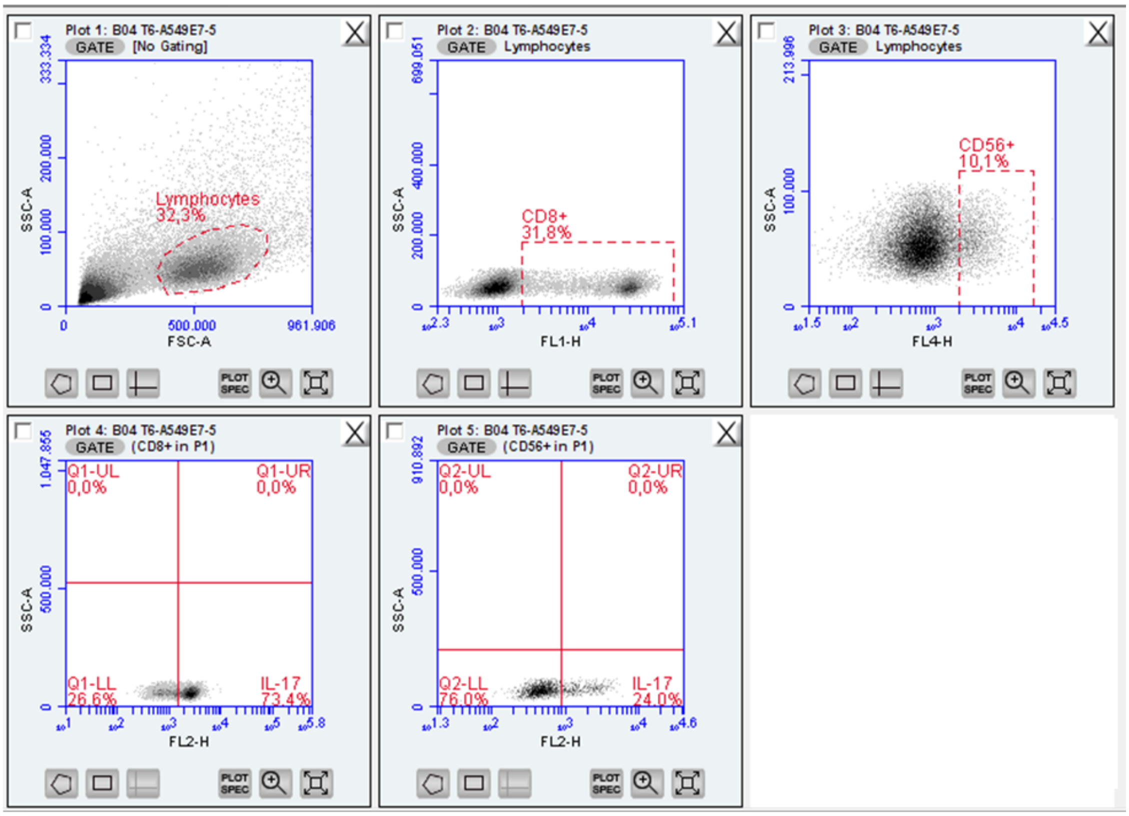

Figure S8. The gating strategy to identify the IL-17 production in CD8+ T cells and CD56+ T cells. CD8+ T cells were identified by FITC staining in the FL1-H channel, while CD56+ cells were stained with APC and detected in the FL4-H channel. IL-17 production was then analyzed within both CD8+ and CD56+ populations using PE staining in the FL2-H channel.

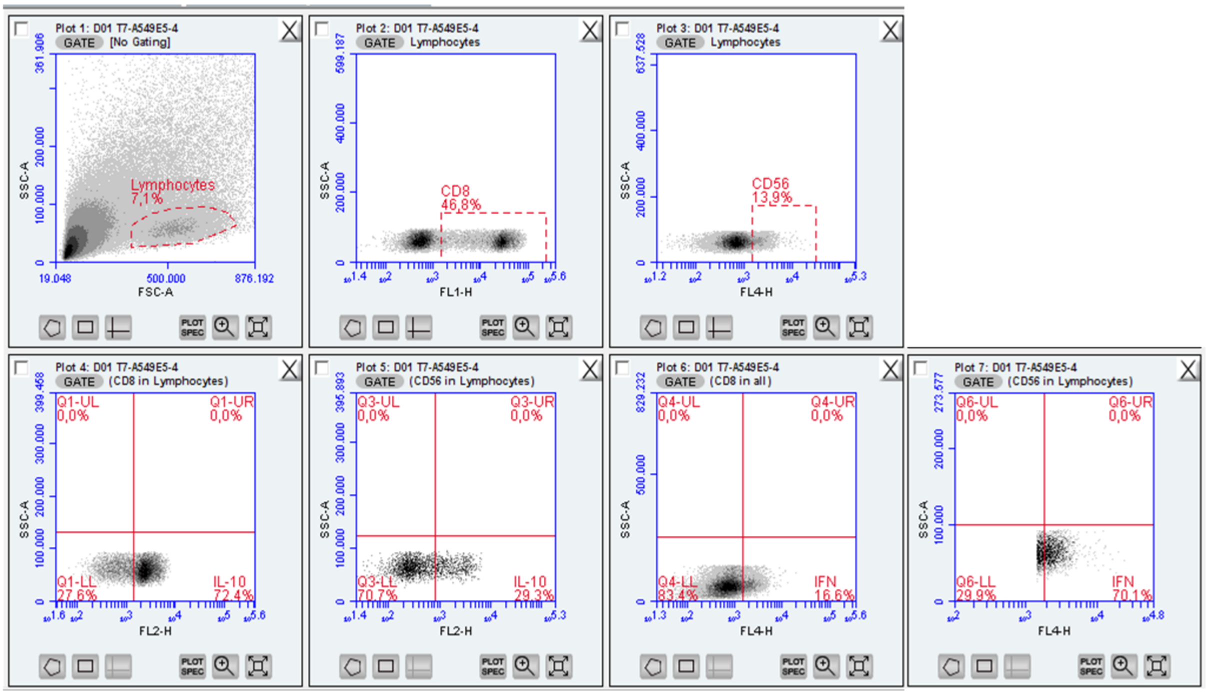

Figure S9. The gating strategy to identify the IL-10 and IFN production in CD8+ T cells and CD56+ T cells. CD8+ T cells were identified by FITC staining in the FL1-H channel, while CD56+ cells were marked with PECy5 and detected in the FL4-H channel. IL-10 expression was evaluated in both CD8+ and CD56+ populations using PE staining in the FL2-H channel, and IFN-γ production was assessed using APC staining in the FL4-H channel.

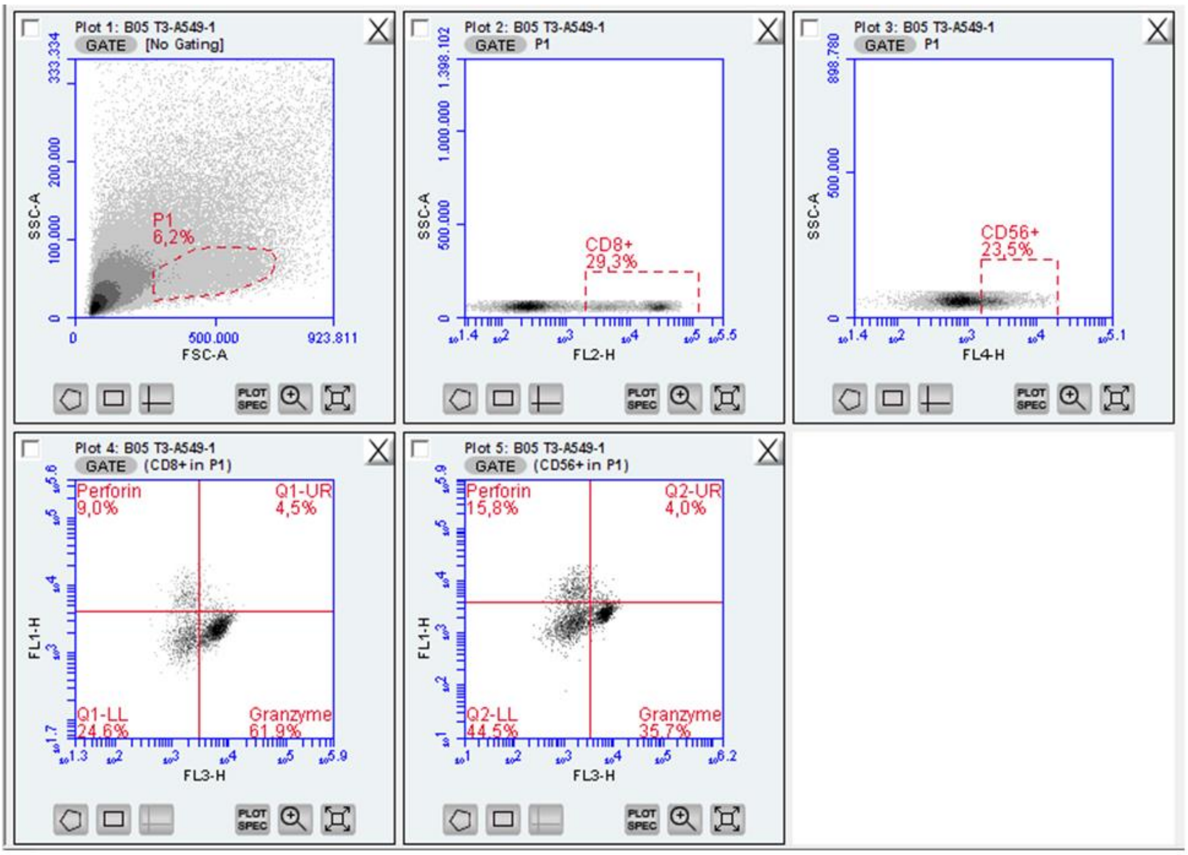

Figure S10. Gating strategy for assessing the cytotoxic profile of CD8+ T cells and CD56+ cells based on intracellular production of granzyme and perforin. CD8+ T cells were identified using PE staining in the FL2-H channel, while CD56+ cells were detected with APC in the FL4-H channel. Intracellular perforin expression was analyzed in both populations using FITC in the FL1-H channel, and granzyme production was measured using PerCP in the FL3-H channel.

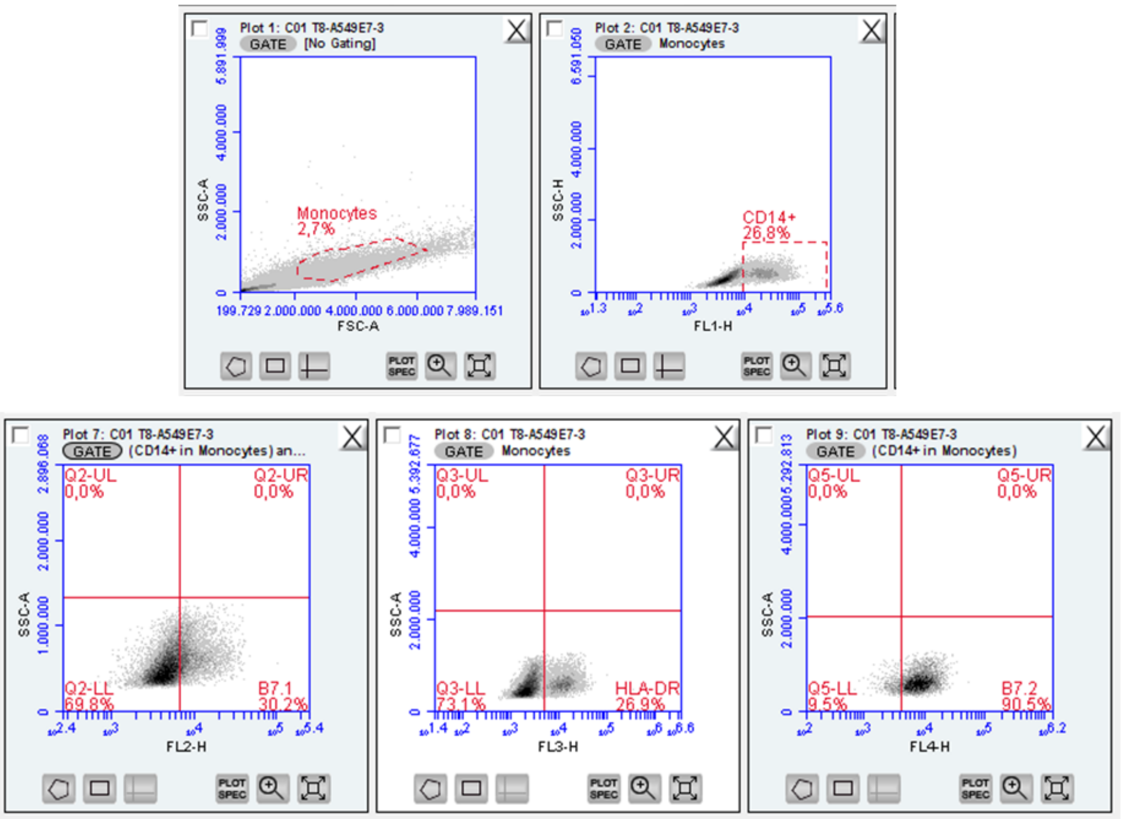

Figure S11. Gating strategy for the characterization of monocytes based on the expression of co-stimulatory molecules on their surface. Monocytes were identified as CD14+ cells using FITC staining, detected in the FL1-H channel. The co-stimulatory molecule B7.1 was labeled with PE and analyzed in the FL2-H channel, while HLA-DR was labeled with PerCP and detected in the FL3-H channel. The co-stimulatory molecule B7.2 was stained with APC and read in the FL4-H channel.

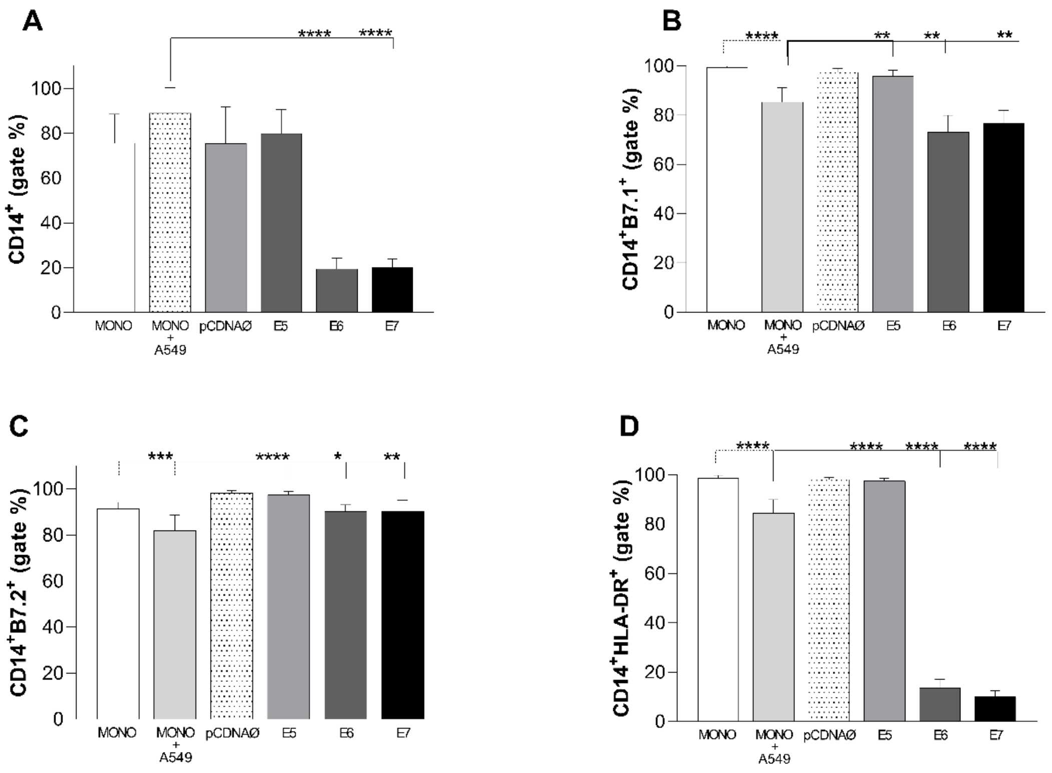

Figure S12. Monocytes CD14^{+} cultured with A549 cell lineage (with or without HPV16 oncogenes presence). **A** – decrease of monocytes number. **B** – decrease of B7.1 expression. **C** – increase of B7.2 expression, and **D** – decrease of HLA-DR expression. The *p* values were obtained comparing the group MONO+A549 with E5, E6, and E7, respectively. *(*p<0.05, **p<0.01, ***p<0,001, ****p<0.0001*).

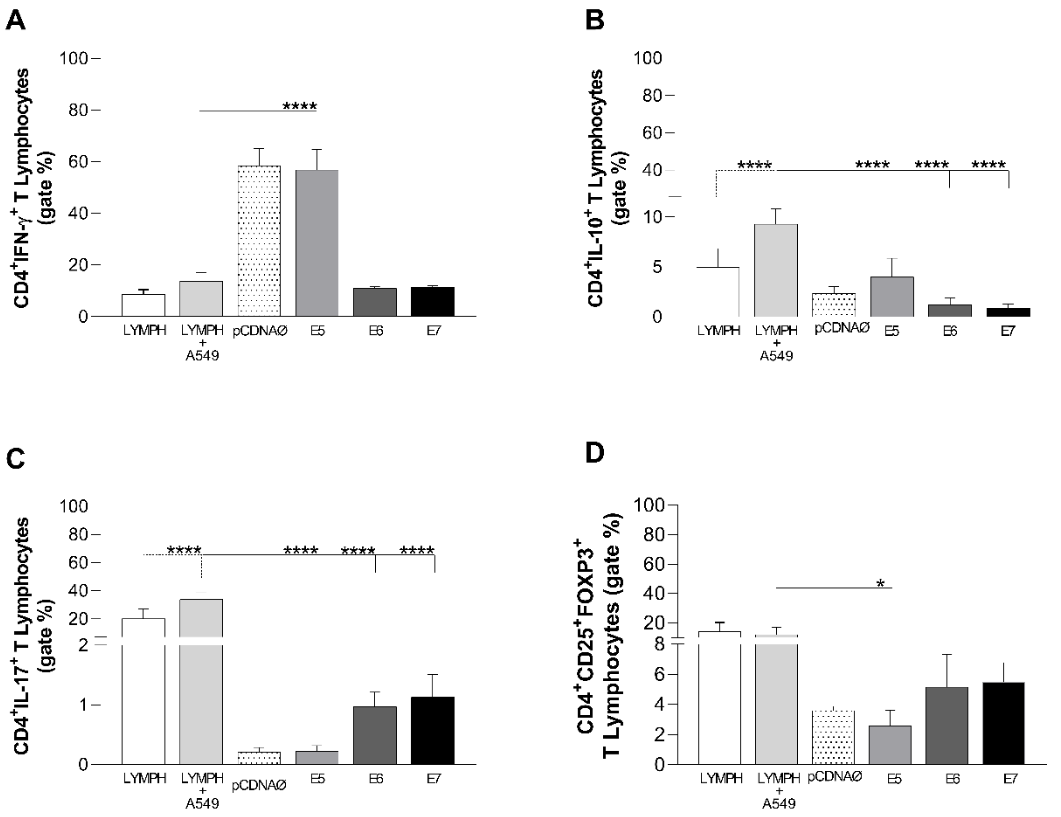

Figure S13. CD4 T lymphocytes phenotype induced by A549 tumor cell lineage. **A**, **B**, and **C** – IFN-γ, IL-10, and IL-17 intracellular production observed in T CD4+ cells, respectively. ****p<0.0001. **D** – CD4 T regulatory cells (CD4+CD25+FOXP3+). *p = <0.01.

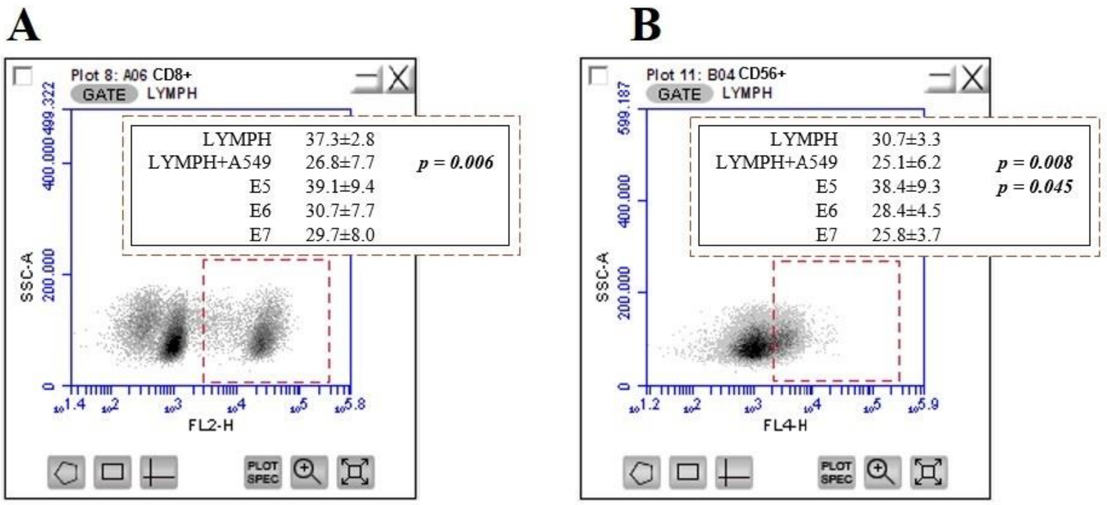

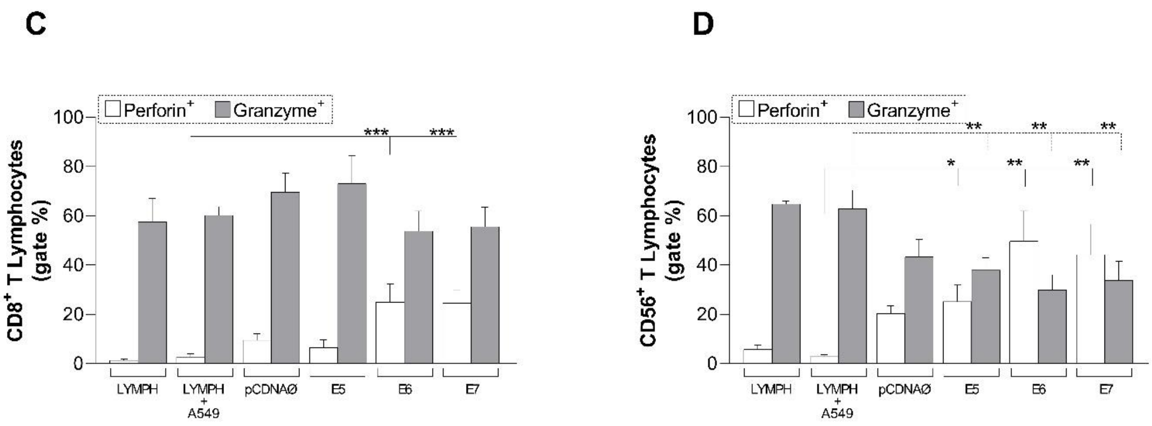

Figure S14. $CD8^+$ and $CD56^+$ lymphocytes stimulation promoted by A549 tumor cells. **A** – Density plot of $CD8^+$ T cells showing the decrease of cells number against A549. **B** - Density plot of $CD56^+$ T cells showing the decrease of cells number against A549 (LYMPH *versus* LYMPH+A549) and increase of cells number in E5 group (LYMPH+A549 *versus* LYMPH+A549E5). **C** and **D** – Perforin and granzyme intracellular production by CD8+ T and CD56+ lymphocytes, respectively. **p = 0.0145*, ***p < 0.005; ***p<0.0003; ****p<0.0001.*

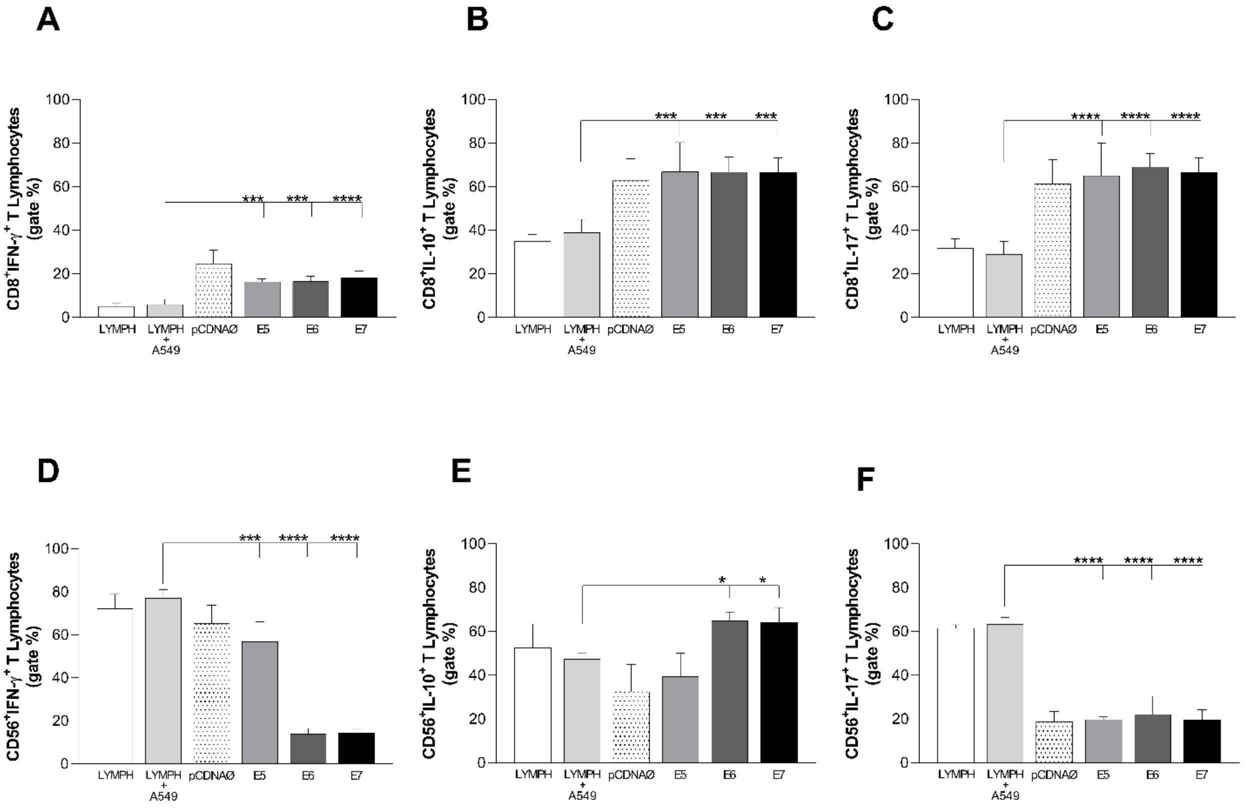

Figure S15: Intracellular cytokines produced by CD8+ T and CD56+ lymphocytes against A549 tumor cells. A, B, and C – IFN-γ, IL-10, and IL-17 produced by CD8+ T lymphocytes,

respectively. D, E, and F – IFN-γ, IL-10, and IL-17 produced by CD56+ lymphocytes, respectively. **p < 0.04, ***p = 0.001, ****p < 0.0001.*

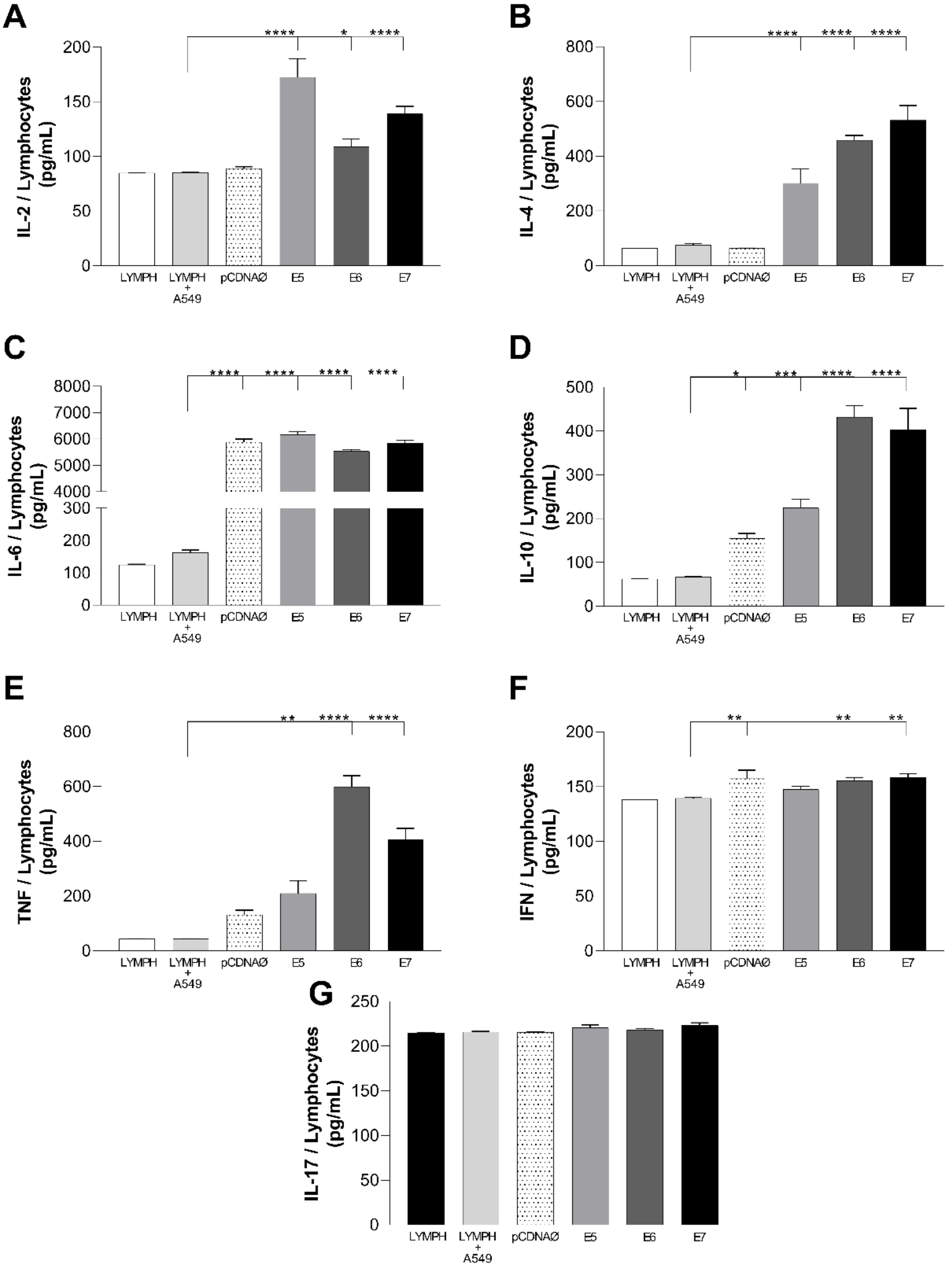

Figure S16. Cytokines present in the supernatant of T lymphocytes cultured with lung tumor cells transfected or not with HPV oncoproteins. A- G – cytokines IL-2, IFN-γ, TNF-α, IL-17, IL-4, IL-10 and IL-6, respectively. Asterisks represent statistical significance *(*p<0.05, **p<0.01, ***p<0.001, ****p<0.0001*). Error bars: standard error between samples.

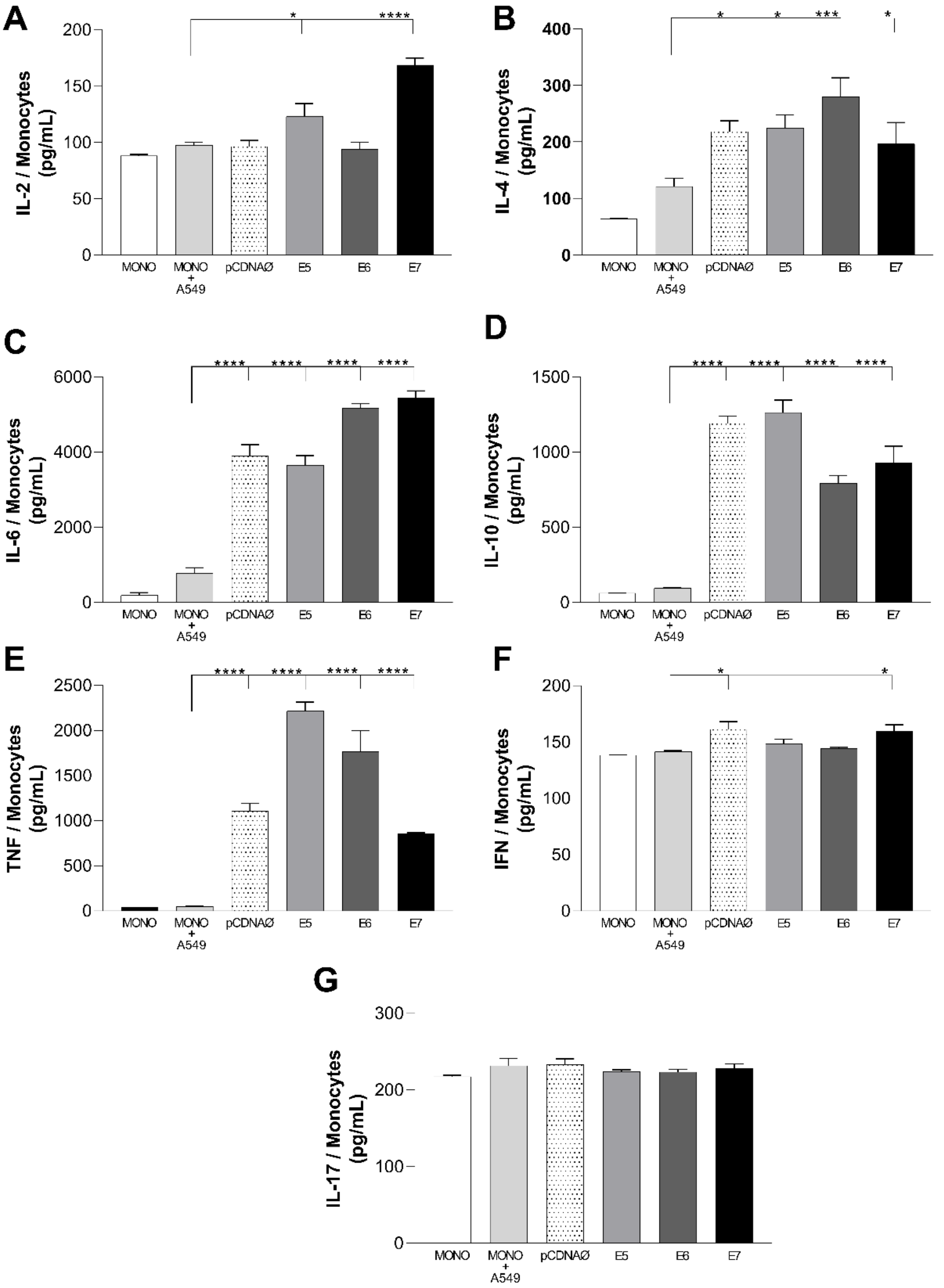

Figure S17. Cytokines present in the supernatant of monocytes cultured with lung tumor cells transfected or not with HPV oncoproteins. A- G – cytokines IL-2, IFN-γ, TNF-α, IL-17, IL-4, IL-10 and IL-6, respectively. Asterisks represent statistical significance *(*p<0.05, **p<0.01, ***p<0.001, ****p<0.0001)*. Error bars: standard error between samples.
